# The First Year Matters: Lifestyle Behaviors and Five-Year Cardiometabolic Risk Factor Accumulation After Traumatic Brain Injury

**DOI:** 10.3390/medsci14020265

**Published:** 2026-05-20

**Authors:** Andrea Calderone, Lilla Bonanno, Fausto Famà, Irene Ciancarelli, Alessio Currò, Angelo Quartarone, Carmela Rifici, Rocco Salvatore Calabrò

**Affiliations:** 1 IRCCS Centro Neurolesi Bonino-Pulejo, S.S. 113 Via Palermo, C.da Casazza, 98124 Messina, Italy; lilla.bonanno@irccsme.it (L.B.); alessio.curro@irccsme.it (A.C.); angelo.quartarone@irccsme.it (A.Q.); carmela.rifici@irccsme.it (C.R.); roccos.calabro@irccsme.it (R.S.C.); 2 Department of Clinical and Experimental Medicine, University of Messina, 98125 Messina, Italy; ffama@unime.it; 3 Department of Life, Health and Environmental Sciences, University of L’Aquila, 67100 L’Aquila, Italy; irene.ciancarelli@univaq.it

**Keywords:** traumatic brain injury, cardiometabolic risk factors, multimorbidity, lifestyle behaviors, rehabilitation, longitudinal cohort, smoking, physical activity

## Abstract

**Background/Objectives:** Traumatic brain injury (TBI) is increasingly understood as a chronic condition, but the role of early post-injury lifestyle behaviors in later cardiometabolic risk remains unclear. We examined whether lifestyle behaviors reported 1 year after injury were associated with the accumulation of common cardiometabolic risk factors by 5 years in the Traumatic Brain Injury Model Systems (TBIMS) National Database. **Methods:** This retrospective cohort secondary analysis included adults with followed 1-year and 5-year interviews, complete 1-year data on four behaviors, and the complete ascertainment of hypertension, diabetes or high blood sugar, and high cholesterol at both waves. The exposure was a favorable lifestyle count based on not smoking, non-heavy alcohol use, non-obese body mass index, and sports or exercise at least 10 times per month. The primary endpoint was the incident accumulation of at least two new common cardiometabolic conditions between years 1 and 5. The analytic cohort was an observed-data subset defined by follow-up retention, complete behavior data, paired outcome ascertainment, and baseline at-risk status rather than a random sample of all TBIMS participants. **Results:** Among 10,057 linked participants with followed interviews at both waves, 9593 were adults, 3182 had complete four-behavior exposure data, 689 had complete cardiometabolic ascertainment, and 581 formed the primary at-risk observed-data cohort. The primary endpoint occurred in 39 participants (6.7%). Each additional favorable behavior was associated with lower odds of the primary endpoint in the adjusted model (odds ratio [OR], 0.63; 95% confidence interval [CI], 0.41–0.98; *p* = 0.040). The results were similar after adjustment for the 1-year Functional Independence Measure cognitive score and in Firth logistic regression. Because the final cohort was selected and the number of primary events was small, the estimates should be interpreted as exploratory and may not generalize to the broader TBI population. **Conclusions:** More favorable 1-year lifestyle profiles were associated with lower 5-year cardiometabolic risk factor accumulation after TBI. These findings support prevention-oriented follow-up but do not establish causality or validate a prognostic score.

## 1. Introduction

Traumatic brain injury (TBI) is increasingly understood as a chronic health condition rather than a single acute neurological event. Beyond the initial injury, many survivors experience long-term changes in functioning, participation, health service use, and general medical vulnerability. This chronic care perspective has shifted attention from neurological recovery alone toward broader post-injury surveillance, including the prevention of potentially modifiable long-term medical risks [1,2,3,4,5,6,7,8,9,10,11,12].

Cardiometabolic conditions are particularly relevant in this context. Prior studies suggest that individuals with a history of TBI may face an increased risk of later cardiovascular, endocrine, and metabolic comorbidity, while multimorbidity more generally is recognized as a major determinant of prognosis, treatment complexity, and health care burden [13,14,15,16,17,18]. Within a TBI rehabilitation cohort, a clinically important question is therefore whether modifiable behaviors observed during recovery are associated with the subsequent accumulation of common cardiometabolic risk factors.

Lifestyle behaviors provide a pragmatic starting point for this question. Cigarette smoking, harmful alcohol use, excess adiposity, and physical inactivity are established cardiometabolic risk domains in the general population, and each is clinically recognizable during follow-up. In the present analysis, the four selected domains, cigarette smoking, alcohol category, body mass index (BMI) category, and sports or exercise frequency, were chosen because they were available at the 1-year TBIMS follow-up, interpretable for clinicians, aligned with broad cardiovascular and metabolic prevention targets, and feasible to review during post-TBI rehabilitation or survivorship care. Other lifestyle determinants, including diet quality, sleep patterns, and medication adherence, are clinically relevant but were not consistently available in a comparable form for the present public-use analytic framework and would have introduced additional construct heterogeneity and missingness. The four-behavior count was therefore intended to summarize a simple observable lifestyle profile, not to create a validated TBI-specific prediction score.

Studying these behaviors after TBI is clinically relevant because survivors may face barriers to sustaining healthy routines, including motor limitations, fatigue, cognitive inefficiency, emotional symptoms, altered social roles, reduced participation, and environmental constraints. Obesity and physical inactivity have been described after TBI, and substance use patterns remain clinically important but heterogeneous. Thus, although the behaviors themselves are not unique to TBI, their measurement within a TBI cohort may help identify prevention-oriented follow-up needs [19,20,21,22].

The Traumatic Brain Injury Model Systems (TBIMS) National Database provides a suitable platform for this type of secondary analysis because it includes standardized baseline characterization and repeated follow-up assessments after inpatient rehabilitation. The database also allows for temporal ordering between 1-year lifestyle exposures and later self-reported medical conditions. However, TBIMS follow-up data include wave-specific missingness, participant-only items, proxy-related limitations, and special codes that require transparent handling. These features make cohort construction, endpoint definition, and sensitivity analyses central to interpretation [23,24,25,26,27,28,29].

The 1-year follow-up was selected as the exposure anchor because it represents a clinically meaningful transition from early post-injury recovery and inpatient rehabilitation toward longer-term community survivorship. By this stage, lifestyle routines, participation patterns, and follow-up needs may be more stable and more clinically reviewable than during the acute or early rehabilitation period. The 5-year follow-up was selected to provide a longer interval over which common cardiometabolic risk factors could accumulate while preserving temporal separation between behavior assessment and subsequent outcome ascertainment. This interval is clinically meaningful because hypertension, diabetes or high blood sugar, and high cholesterol are common, modifiable risk states that may emerge during long-term TBI follow-up and can inform prevention-oriented surveillance.

The present study examined whether a pragmatic 1-year favorable lifestyle behavior count was associated with the accumulation of common cardiometabolic risk factors by 5 years after TBI. The exposure was limited to four observable domains: not smoking, non-heavy alcohol use, non-obese BMI category, and sports or exercise at least 10 times per month. The primary endpoint was defined as the incident accumulation of at least two new common cardiometabolic conditions, hypertension, diabetes or high blood sugar, and high cholesterol, between years 1 and 5. We hypothesized that a higher number of favorable lifestyle behaviors at 1 year would be associated with lower odds of subsequent cardiometabolic risk factor accumulation while recognizing that this association would not establish causality or validate a prognostic tool.

## 2. Materials and Methods

### 2.1. Study Design, Data Source, and Ethical Considerations

This study was a retrospective cohort secondary analysis of de-identified public-use TBIMS data. TBIMS enrolls individuals with moderate-to-severe TBI who receive inpatient rehabilitation at participating U.S. centers and follows them longitudinally with standardized forms. The present analysis used the publicly available Form 1 baseline file, which contains demographic and acute injury variables, together with Form 2 follow-up data, which contain interview-based medical and behavioral measures at scheduled post-injury waves. Records were linked by a unique participant identifier (Mod1Id). Because the analysis used de-identified public-use data, no additional participant contact occurred. This study was conducted in accordance with the ethical principles of the Declaration of Helsinki and with the governance framework applicable to the original TBIMS data collection [30,31,32].

The analytic strategy was driven by three prespecified principles. First, exposure and outcome ordering had to remain temporally clear, so lifestyle behaviors were anchored at the 1-year follow-up, and incident cardiometabolic outcomes were defined over the interval from year 1 to year 5. Second, the primary endpoint needed to be clinically coherent rather than a mixture of conditions with markedly different stages and trajectories. Third, the handling of missingness had to distinguish structural unavailability and clinically informative special codes from ordinary item nonresponse. These principles informed both the cohort construction and the choice of primary and sensitivity analyses.

### 2.2. Eligibility Criteria and Cohort Construction

The source population comprised all available Form 1 baseline records in the public-use dataset. Eligibility required a followed Form 2 interview at both 1 year and 5 years after injury, with follow-up status defined according to the TBIMS interview status code and exposure and outcome ascertainment restricted to records classified as followed at the target waves. Records were linked by a unique participant identifier (Mod1Id), and cohort counts were anchored to participants linkable to a Form 1 baseline record and to followed Form 2 interviews at both target waves. The primary analytic cohort was limited to participants aged 18 years or older at injury to maintain age definition consistency across this study. A prespecified sensitivity analysis repeated the main adjusted model in the broader subgroup aged 16 years or older to reflect the historical TBIMS eligibility framework, which includes late adolescents.

After linkage and age restriction, the next step required valid one-year values for the four primary behavior components: cigarette smoking, alcohol consumption, body mass index category, and sports or exercise frequency. Because the endpoint depended on change between year 1 and year 5, participants also had to have complete values for hypertension, diabetes or high blood sugar, and high cholesterol at both waves. Finally, to preserve a genuine incident multimorbidity framework, the primary at-risk cohort excluded participants who already had two or more of the three common cardiometabolic conditions at year 1. Participants with zero or one baseline condition remained eligible because both groups could still develop two newly incident common cardiometabolic conditions over the interval. Participants with no common condition at year 1 were classified as primary endpoint cases if they developed at least two new conditions by year 5, whereas participants with one common condition at year 1 were classified as primary endpoint cases only if they developed two additional new conditions by year 5. Participants who developed exactly one new condition were captured by the secondary endpoint, not the primary endpoint.

The resulting cohort was progressively reduced across successive eligibility and data completeness requirements. This pattern reflects the structure of TBIMS follow-up data, in which some variables are available only under specific interview conditions, and certain participant-reported items may be unavailable when follow-up information is obtained by proxy. Accordingly, the final primary cohort should be interpreted as a selected observed-data subset rather than as a fully representative sample of all linked TBIMS participants or all individuals living with TBI. To preserve interpretability, the primary adjusted model used a deliberately parsimonious complete case approach after the at-risk cohort had been defined from observed exposure and outcome data, whereas broader patterns of inclusion were described separately to contextualize the final analytic sample.

The cohort restrictions were therefore treated as an analytic feature requiring interpretation rather than as routine attrition. Participants excluded because of unavailable behavior items, invalid cardiometabolic responses, proxy-related participant-only items, or missing covariates could differ from included participants in functional status, health care access, health surveillance, lifestyle pattern, or cardiometabolic burden. For this reason, the final analyses and interpretation distinguish the internal association estimated within the complete observed-data cohort from the broader question of transportability to all TBIMS participants or to all people living after TBI.

### 2.3. Outcome Definitions and Measures

The primary outcome was incident cardiometabolic risk factor multimorbidity between 1 and 5 years after injury. This was defined as the development of at least two new common cardiometabolic conditions among hypertension, diabetes or high blood sugar, and high cholesterol. Operationally, the endpoint counted newly incident conditions only, not total year-5 burden. Participants with no common cardiometabolic condition at year 1 were primary endpoint cases if they developed at least two of the three conditions by year 5. Participants with one common condition at year 1 were primary endpoint cases only if they developed both of the remaining two conditions by year 5. Participants who developed exactly one new condition were counted in the secondary endpoint, and participants who already had two or more common conditions at year 1 were excluded from the primary at-risk cohort. These three conditions were chosen because they are common clinical risk states and are directly relevant to later cardiovascular disease, and it is plausible for them to accumulate over the follow-up interval available in TBIMS. Each variable was taken from the corresponding physician-diagnosed condition item on Form 2 and coded as present or absent using the valid TBIMS response categories. Participants with special codes indicating unknown, refused, or otherwise invalid status at either wave for any of the three conditions were not eligible for the primary endpoint because incident status could not be determined reliably.

The secondary endpoint captured any common cardiometabolic risk factor incidence and was defined as the development of at least one new condition among the same three domains between years 1 and 5. Three condition-specific secondary outcomes were also examined separately: incident hypertension, incident diabetes or high blood sugar, and incident high cholesterol. For these models, each analysis was restricted to participants free of the corresponding condition at year 1 and with valid ascertainment at both year 1 and year 5. These outcome-specific analyses were intended to clarify whether the primary association was distributed broadly across domains or driven primarily by one component.

The broader six-condition composite consisted of hypertension, diabetes or high blood sugar, and high cholesterol along with myocardial infarction, congestive heart failure, and stroke. This broader six-condition endpoint was retained only as a sensitivity analysis. It was not used as the primary outcome because it combines common risk factors with less frequent and clinically distinct vascular events whose natural history and short-term incidence are not directly comparable. The primary outcome hierarchy therefore placed the common three-condition accumulation endpoint first while allowing the six-condition composite to remain available for transparency and comparability. This hierarchy is more consistent with prevention-oriented cardiovascular frameworks and with the logic of incident risk factor accumulation [33,34,35,36].

### 2.4. Exposure Definitions

The primary exposure was a one-year favorable lifestyle behavior count ranging from 0 to 4. One point was assigned for each of the following at the 1-year interview: not smoking cigarettes at all, non-heavy alcohol use, non-obese body mass index category, and participation in sports or exercise at least 10 times per month. This summary was selected because the four component behaviors are clinically interpretable, feasible to obtain in routine follow-up, and sufficiently independent in meaning to support a parsimonious cumulative count. The equal one-point weighting was a pragmatic and transparent summary choice intended to reduce analytic complexity and improve communication; it was not intended to assert that smoking, alcohol pattern, adiposity, and exercise have identical biological effects, identical causal relevance, or identical prognostic weight after TBI. The count was treated as a continuous exposure in the main models, thereby estimating the change in odds associated with each additional favorable behavior. For descriptive presentation, the distribution was also summarized in ordered categories of 0 to 1, 2, 3, and 4 favorable behaviors.

Diet quality, sleep patterns, medication adherence, and other health management behaviors were not included in the primary count. These domains are clinically important, but they were not consistently available as comparable, valid, and temporally aligned variables within the public-use analytic dataset used for this study. Including them would also have broadened the construct from observable lifestyle behaviors to a more heterogeneous health management index and would likely have increased missingness in an already selected analytic cohort. These factors were therefore treated as potential unmeasured or residual confounders rather than as components of the primary exposure.

Smoking status was derived from the Form 2 cigarette use item and classified for the primary count as favorable only when the participant reported not smoking at all. Alcohol use was derived from the Form 2 consumption category item and treated as favorable when the reported category was abstaining, light, or moderate rather than heavy. Body mass index was taken from the categorized Form 2 BMI variable and treated as favorable when the participant was not in an obesity category. Exercise was derived from monthly sports or exercise frequency and treated as favorable when participation occurred at least 10 times per month. These threshold choices were designed to preserve a clinically transparent summary aligned with broad cardiovascular prevention principles while remaining faithful to the response structure available in the TBIMS public-use data [37,38,39,40,41,42,43].

To preserve conceptual separation between perceived health and observable behaviors, self-rated general health was intentionally excluded from the primary lifestyle count. Instead, general health was examined separately as a distinct one-year contextual variable. It was first modeled alone on its available subset and then entered jointly with the four-behavior count in an exploratory model restricted to participants with valid general health data. This allowed for an assessment of whether perceived overall health simply mirrored the behavior count or captured an additional signal more consistent with global illness burden or residual disability.

To avoid overreliance on a simple summary index, component-level models were also prespecified. Smoking, alcohol, BMI, and exercise were each examined in collapsed multi-level categories chosen before modeling to preserve clinically meaningful contrast while avoiding overly sparse cells. These component models addressed the conceptual concern that equal weighting in a count might conceal marked heterogeneity among domains. The component results were interpreted as exploratory and hypothesis-generating rather than as separate confirmatory tests of individual behavior effects.

### 2.5. Covariates, TBI-Related Variables, and Data Handling

The primary adjusted model included age at injury, sex, race or ethnicity, education, and the year-1 baseline burden of the three common cardiometabolic conditions. Age at injury was drawn from the TBIMS non-protected age variable. Sex was taken from the Form 1 sex variable. Race or ethnicity was collapsed into White, Black, Hispanic, and Other categories to support stable estimation. Education was grouped into high school or trade school or less, some college or associate degree, and a bachelor’s degree or higher; a small unknown category was retained descriptively and excluded only if required for complete case adjusted modeling. Baseline cardiometabolic burden at year 1 was categorized as zero versus one of the three common conditions. This variable was included because the clinical meaning of a new event differs depending on whether participants already carried one common risk factor at the start of the interval.

Injury severity variables were treated with more nuance than a default complete case approach. The Glasgow Coma Scale (GCS) category was not forced into the primary model because public-use coding includes a clinically meaningful intubated or unassessable state, and the linked data showed substantial nonstandard or missing coding in this field. Instead, the GCS was evaluated in a dedicated sensitivity model with explicit categories for severe, moderate, mild, intubated, and missing or unknown. Post-traumatic amnesia (PTA) duration was similarly handled with clinically interpretable groupings that preserved the code indicating a status of still in PTA at rehabilitation discharge rather than absorbing it into generic missingness. These design choices were intended to respect the informational content of TBIMS special codes and to avoid the misleading assumption that all unavailable severity data are exchangeable [44,45,46].

At least one post-injury TBI-relevant sequela variable was also considered necessary because lifestyle behaviors are not inherently TBI-specific. The year-1 Functional Independence Measure cognitive subscale score was chosen as the main contextual sequela variable for robustness testing because it was clinically interpretable, reasonably complete in the target cohort, and plausibly related to both post-injury health behavior and later medical vulnerability. A robustness model therefore added the year-1 cognitive score to the primary adjusted covariate set. This did not convert the model into a causal mediation analysis; rather, it examined whether the observed association between the behavior count and subsequent cardiometabolic accumulation remained materially similar after accounting for a broad marker of residual post-injury functional status.

The handling of missing data followed the structure of the data rather than a single universal rule. Exposure and outcome variables were not imputed because large portions of missingness reflected structural unavailability or wave-specific invalidity that could not be assumed missing at random in a defensible way. For example, participant-only items may be absent when a proxy completed the interview, and special codes may reflect interview circumstances rather than stochastic missingness. Covariate missingness in the final adjusted model was minimal after the at-risk cohort had been defined; accordingly, the primary adjusted analyses used complete case covariates, whereas broader cohort selection and completeness patterns were documented descriptively. These restrictions may introduce selection bias if the availability of behavior data, outcome ascertainment, or covariates was related to both the one-year lifestyle profile and later cardiometabolic status. The complete case strategy was therefore paired with explicit cohort flow reporting, included-versus-excluded comparisons, and cautious generalizability language rather than being treated as a neutral analytic convenience. This approach prioritized transparency and reproducibility over an imputation strategy that would have required stronger assumptions than the structure of the public-use file could justify. Descriptive frequencies were calculated in the full at-risk cohort. Regression models were then estimated in model-specific complete case samples defined by the availability of all covariates required for the given model; therefore, model denominators and event counts can differ slightly from the descriptive frequencies reported in the main tables.

### 2.6. Statistical Analysis

Descriptive statistics were used to summarize cohort assembly, baseline characteristics, exposure distributions, baseline cardiometabolic burden, and incident outcome frequencies. Continuous variables are presented as the mean with standard deviation and categorical variables as a number with percentage. The descriptive characteristics of the primary at-risk cohort were summarized overall and by primary endpoint status. A separate comparison between the final analytic cohort and eligible participants excluded during later cohort construction was prepared using standardized mean differences to characterize potential selection patterns without overemphasizing significance testing in a highly imbalanced comparison. The primary endpoint occurred in 39 of 581 participants (6.7%) in the descriptive at-risk cohort, whereas the primary adjusted model was fit in 577 participants with 38 events because four participants had missing sex or education data. The corresponding model, additionally adjusted for the year-1 FIM cognitive score, included 565 participants with 37 events because 12 additional participants lacked valid FIM cognitive data. For the secondary endpoint, 162 of 581 participants (27.9%) met the descriptive definition, whereas the adjusted complete case model included 577 participants with 160 events.

The main inferential analysis used logistic regression to estimate the association between the one-year favorable lifestyle behavior count and the primary endpoint. Model 1 was unadjusted. Model 2 adjusted for age at injury, sex, race or ethnicity, education, and baseline common cardiometabolic burden. Model 3 added the year-1 FIM cognitive score to the Model 2 covariates. The odds ratio was expressed per one-point increase in the favorable behavior count. Because the event rate was modest and sparse data vulnerability remained a central analytic consideration, the covariate set was intentionally restricted to a small number of clinically plausible confounders rather than an exhaustive baseline inventory. Given the small number of primary events, the primary analysis was interpreted as exploratory, with emphasis placed on effect direction, confidence intervals, and robustness rather than on the *p* value alone.

The potential non-linearity of age was evaluated formally by comparing the primary adjusted model containing linear age with an otherwise identical model containing restricted cubic spline terms for age. Because the likelihood ratio comparison did not support non-linearity, age was retained as a linear term in the main presentation. This step was included to address the possibility that age-related selection into the analytic cohort or age-related cardiometabolic risk might have been inadequately represented by a linear term. The design analysis supplementary material also reports the observed number of events per parameter and an approximate detectable effect size under the final sample and event structure to contextualize precision limitations.

Several robustness analyses were prespecified. First, a Firth bias-reduced logistic regression version of the primary adjusted model was fit to evaluate whether sparse data bias materially altered the main inference. Second, the primary adjusted model was repeated in the broader cohort of those aged 16 years or older. Third, separate sensitivity models added explicit GCS categories and explicit PTA categories to the primary adjusted covariate set. Formal severity-stratified models were not pursued because the primary endpoint was sparse, and stratification by GCS or PTA categories would have produced unstable subgroup estimates, particularly given special and missing injury severity codes in the public-use dataset. The GCS- and PTA-adjusted models were therefore treated as severity-coding sensitivity analyses rather than as tests of effect modification by injury severity. Fourth, the original six-condition mixed composite was reanalyzed in an adjusted model using an appropriate baseline six-condition burden term. Finally, the secondary endpoint of any incident common cardiometabolic condition and the three individual condition-specific outcomes were modeled to clarify the domain-specific pattern of association; these condition-specific models were restricted to participants free of the index condition at year 1 and were adjusted for age, sex, race or ethnicity, and education. An additional ordinal logistic regression sensitivity analysis modeled the ordered number of incident common cardiometabolic conditions as 0, 1, or at least 2 using the same covariate set as the primary adjusted model. Because the proportional odds framework is more assumption-dependent and the highest-burden category remained sparse, this model was treated as a sensitivity analysis rather than as a replacement for the prespecified binary primary endpoint. Time-to-event methods were not used because the TBIMS condition variables provide status at scheduled follow-up waves rather than exact dates of diagnosis or onset; therefore, the data support wave-based interval comparisons rather than survival modeling.

All analyses were performed from the original public-use dataset files after verifying variable coding against the TBIMS data dictionary, auxiliary code files, variable lists, and available SPSS (version 4.4.2; R Foundation for Statistical Computing, Vienna, Austria) syntax files. Statistical significance was interpreted cautiously and always alongside the point estimate and confidence interval. The analyses were reported according to the Strengthening the Reporting of Observational Studies in Epidemiology statement. Supplementary Material S1 documents cohort assembly; Supplementary Material S2 compares included and excluded participants; Supplementary Material S3 provides the analytic codebook, recoding rules, and reproducibility pseudocode; Supplementary Material S4 details wave-specific completeness; Supplementary Material S5 summarizes sparse data contextualization and the age form assessment; Supplementary Material S6 reports robustness analyses; Supplementary Material S7 reports alternative endpoint models, ordinal sensitivity analysis, and model-based predicted probabilities; and Supplementary Material S8 contains the final STROBE checklist [47,48,49,50,51,52].

During manuscript preparation, the latest available version of ChatGPT (OpenAI, GPT-5.5 Thinking model, ChatGPT web interface) was used for language editing support, figure creation, visual layout, and graphical formatting. No artificial intelligence tool replaced authorial judgment, and the authors remain fully responsible for the content of the manuscript, analyses, and figures.

## 3. Results

**Figure 1:**
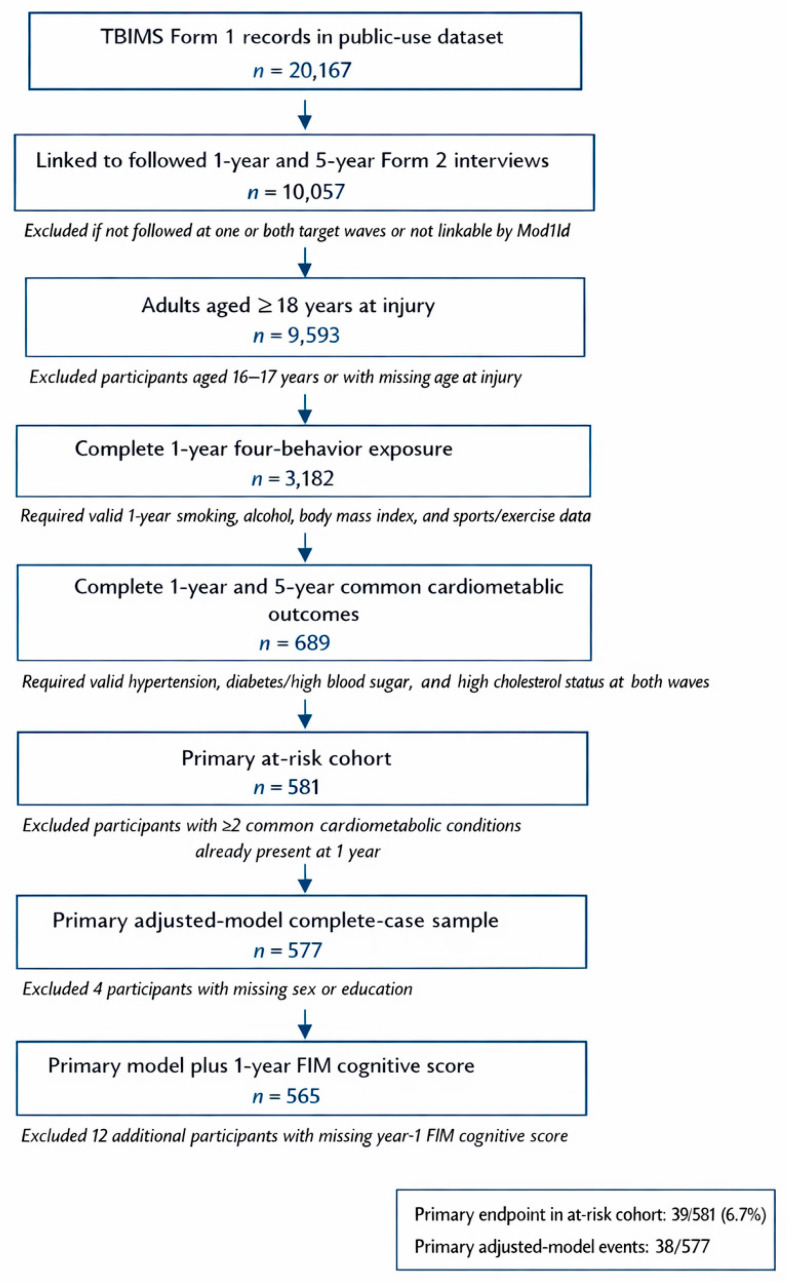
Cohort assembly for the primary analysis. This flow diagram shows the derivation of the adult analytic cohort from the Traumatic Brain Injury Model Systems public-use dataset. Participants were required to have followed interviews at 1 and 5 years, complete 1-year four-behavior exposure data, and the complete ascertainment of the three common cardiometabolic conditions at both waves. This figure also distinguishes the primary at-risk cohort, the adjusted-model sample, and the subset additionally analyzed with the 1-year Functional Independence Measure cognitive score.

The cohort assembly process began with 20,167 Form 1 records in the public-use file. Of these, 10,057 participants could be linked to followed Form 2 interviews at both 1 year and 5 years after injury. Restricting the analysis to adults aged 18 years or older yielded 9593 eligible participants. Requiring complete one-year information for the four primary behavior components reduced the cohort to 3182 participants, and requiring a valid ascertainment of hypertension, diabetes or high blood sugar, and high cholesterol at both year 1 and year 5 reduced it further to 689 participants. Excluding those who already had two or more of the three common cardiometabolic conditions at year 1 resulted in a primary at-risk cohort of 581 participants. The primary adjusted model included 577 participants after the exclusion of four individuals with missing sex or education data, and the model additionally adjusting for the year-1 FIM cognitive score included 565 participants. Baseline differences between included and later excluded eligible participants were generally modest, with the largest standardized mean differences observed for Hispanic ethnicity and missing GCS category rather than for age or sex. The cohort characteristics of the primary at-risk sample are shown in Table 1. Figure 1 shows the derivation of the primary analytic cohort.

The primary at-risk cohort had a mean age at injury of 39.8 (17.7) years, and 146 (25.1) were female. There were 364 White participants (62.7), 102 Black participants (17.6), 90 Hispanic participants (15.5), and 25 participants in the Other category (4.3). More than half of the cohort had a high school or trade school education or less, and most participants entered the interval with no common cardiometabolic conditions at year 1. The mean year-1 FIM cognitive score was 30.6 (5.4). Injury severity descriptors were heterogeneous, but the descriptive tables also highlighted the importance of special coding in the GCS and PTA, especially the nontrivial frequency of missing or clinically informative severity codes that would have been obscured by a naive complete case approach.

Exposure distributions and endpoint frequencies are summarized in Table 2. The favorable lifestyle count was concentrated in the middle and upper range: 24 participants (4.1%) had 0 to 1 favorable behaviors, 126 (21.7%) had 2, 288 (49.6%) had 3, and 143 (24.6%) had all 4. Daily smoking was present in 91 participants (15.7%), while 190 participants (32.7%) reported no sports or exercise participation. The primary endpoint of two or more incident common cardiometabolic conditions occurred in 39 of 581 participants (6.7%). The secondary endpoint of at least one incident common condition occurred in 162 of 581 participants (27.9%). In the broader 689-participant cohort with complete common risk factor ascertainment, hypertension was present in 31.2% at year 1 and 44.7% at year 5, with 96 incident cases among the 474 participants at risk. High cholesterol rose from 19.4% at year 1 to 31.6% at year 5, with 86 incident cases among 555 participants at risk. Diabetes or high blood sugar rose from 8.1% to 13.5%, with 37 incident cases among 633 participants at risk. These descriptive findings indicate that the accumulation of common risk factor states was clinically meaningful over the observed interval even within a relatively young rehabilitation cohort.

### Association Analyses and Robustness Checks

**Figure 2:**
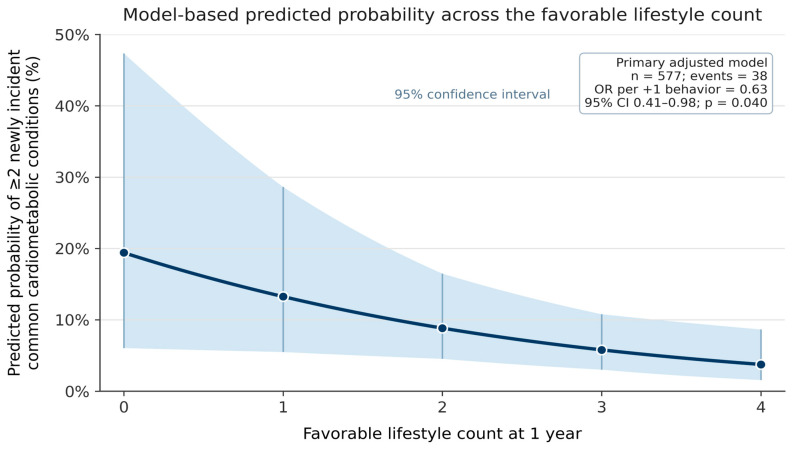
The model-based predicted probability of the primary endpoint across the 1-year favorable lifestyle count. Predicted probabilities were derived from the primary adjusted logistic regression model for developing at least two newly incident common cardiometabolic conditions between years 1 and 5, with lifestyle counts ranging from 0 to 4. Shaded bands indicate 95% confidence intervals. Covariates were held at representative values from the primary adjusted model sample: age 39.7 years, male sex, White race/ethnicity, high school/trade education or less, and no common cardiometabolic condition at year 1. The predicted probabilities decreased from 19.4% at a count of 0 to 3.8% at a count of 4, but the confidence interval was especially wide for the sparsely populated count-0 category. This figure is intended to aid the absolute risk interpretation of the adjusted association and should not be used as a validated individual prediction tool.

The main regression results are shown in Table 3. For direct comparability with the primary adjusted model, the unadjusted estimate was calculated in the same complete case sample. In this unadjusted model, each additional favorable lifestyle behavior at year 1 was associated with lower odds of the primary endpoint, although the estimate was imprecise and did not reach conventional statistical significance (odds ratio 0.72, 95% confidence interval 0.48–1.07; *p* = 0.100). In the primary adjusted model, the association strengthened modestly: each additional favorable behavior was associated with 37% lower odds of developing at least two new common cardiometabolic conditions between years 1 and 5 (odds ratio 0.63, 95% confidence interval 0.41–0.98; *p* = 0.040). Because only 38 primary events were available in the adjusted complete case model and the confidence interval approached the null, this finding should be interpreted as exploratory, statistically borderline, and imprecise rather than confirmatory. The addition of the year-1 FIM cognitive score yielded a nearly identical estimate (odds ratio 0.64, 95% confidence interval 0.41–0.99; *p* = 0.047), suggesting that the association was not explained solely by broad cognitive functional status at one year. The secondary endpoint of at least one incident common condition showed a similar but slightly smaller association (odds ratio 0.74, 95% confidence interval 0.58–0.95; *p* = 0.020), with the estimate again remaining stable after FIM cognitive adjustment (odds ratio 0.73, 95% confidence interval 0.56–0.94; *p* = 0.016). By contrast, the sensitivity analysis using the original six-condition composite produced a directionally similar but statistically imprecise estimate (odds ratio 0.76, 95% confidence interval 0.52–1.10; *p* = 0.143). The ordinal logistic sensitivity analysis, which modeled 0, 1, or at least 2 incident common cardiometabolic conditions, included 577 participants distributed as 417, 122, and 38 across the three ordered categories; the adjusted proportional odds estimate was directionally consistent with the primary analysis (odds ratio 0.72 per additional favorable behavior, 95% confidence interval 0.57–0.92; *p* = 0.010). A descriptive threshold-specific comparison showed a similar direction for the ≥1-condition threshold and the ≥2-condition threshold, supporting sensitivity interpretation while reinforcing that the binary primary endpoint remained the prespecified clinical accumulation threshold. Figure 2 summarizes the model-based predicted probability of the primary endpoint across the favorable lifestyle count.

**Figure 3:**
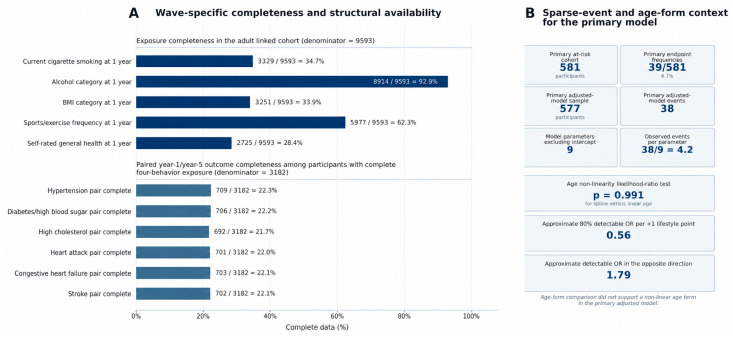
Data completeness and analytic context summary. Panel (**A**) summarizes observed data completeness for the 1-year exposure variables in the adult linked cohort and paired year-1/year-5 ascertainment for the cardiometabolic outcomes among participants with complete four-behavior exposure data. Panel (**B**) summarizes the sparse event context of the primary adjusted model, including the at-risk cohort size, event count, number of model parameters, observed events per parameter, age form assessment, and approximate detectable odds ratio context. Together, these data provide methodological context for the complete case modeling strategy and the cautious interpretation of the primary association estimates.

The formal assessment of age specification did not support meaningful non-linearity; the likelihood ratio comparison between linear and spline age terms yielded *p* = 0.991, so linear age was retained in the main model. The sparse data design analysis showed that the primary adjusted model contained 38 events across nine non-intercept parameters, corresponding to 4.2 observed events per parameter, which reinforced the importance of parsimonious covariate selection and sensitivity analysis. The Firth bias-reduced logistic regression version of the primary adjusted model yielded an odds ratio of 0.64 (95% confidence interval 0.42–0.97; *p* = 0.037), closely matching the standard maximum likelihood estimate. The sensitive analysis of those aged 16 years or older also remained very similar (odds ratio 0.63, 95% confidence interval 0.41–0.97; *p* = 0.037). When explicit GCS categories were added, the estimate was unchanged in direction and magnitude (odds ratio 0.63, 95% confidence interval 0.41–0.98; *p* = 0.041). When explicit PTA categories were added, the estimate remained similar but slightly less precise (odds ratio 0.65, 95% confidence interval 0.42–1.00; *p* = 0.052). Taken together, these results suggest that the main finding was reasonably stable to several analytically relevant perturbations while still reflecting a modest event count and therefore limited precision. The ordinal model and the predicted probability display provide complementary interpretation but do not convert the lifestyle count into a calibrated prediction instrument. Figure 3 summarizes sparse data contextualization and the age form assessment.

**Figure 4:**
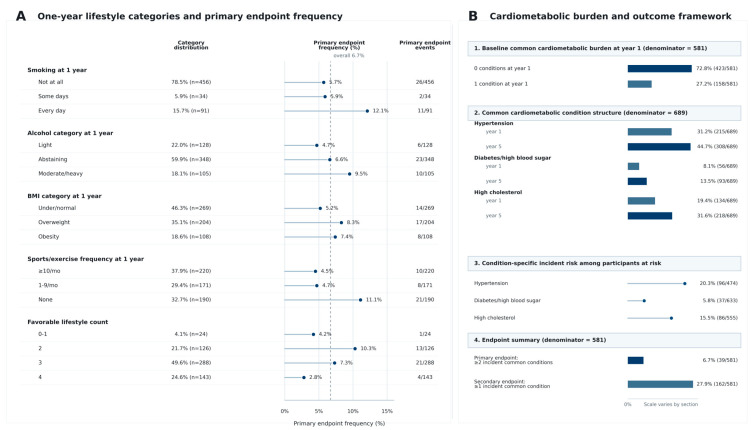
One-year lifestyle categories and a cardiometabolic outcome framework. Panel (**A**) shows the distribution of the 1-year lifestyle categories in the primary at-risk cohort together with the corresponding frequency of the primary endpoint within each category. Panel (**B**) summarizes baseline common cardiometabolic burden at year 1; the year-1 and year-5 prevalence of hypertension, diabetes/high blood sugar, and high cholesterol; condition-specific incident risk among participants at risk; and the frequencies of the primary and secondary endpoints. This figure intends to clarify both the exposure profile of the cohort and the descriptive structure of the cardiometabolic outcome framework.

Component-level and general health analyses are shown in Table 4. Within the component models, daily smoking was associated with higher odds of the primary endpoint compared with not smoking at all (odds ratio 3.33, 95% confidence interval 1.38–8.02; *p* = 0.007), whereas smoking on some days was not clearly different from not smoking. Participants reporting no sports or exercise had higher odds of the primary endpoint than those exercising at least 10 times per month (odds ratio 2.45, 95% confidence interval 1.06–5.68; *p* = 0.037), while intermediate exercise frequency was not clearly separated from the reference category. These smoking and exercise estimates were based on sparse primary event counts within component categories and should be treated as hypothesis-generating signals, not as definitive evidence that these domains alone explain the overall lifestyle count association. Collapsed alcohol and BMI categories showed directionally less favorable estimates for moderate or heavy drinking and for excess body weight, but confidence intervals were wide and crossed the null. Outcome-specific models suggested that the strongest association of the four-behavior count was with incident diabetes or high blood sugar (odds ratio 0.53, 95% confidence interval 0.34–0.83; *p* = 0.005), while associations with incident hypertension and incident high cholesterol were directionally protective but imprecise. Self-rated general health showed a graded association with the primary endpoint when analyzed separately, with fair or poor health associated with substantially higher odds than excellent or very good health. In a joint model on the restricted subset with valid general health data, the self-rated health gradient remained more prominent than the behavior count itself, underscoring the conceptual importance of keeping perceived health separate from the primary lifestyle summary rather than embedding it within the main exposure. Figure 4 provides a descriptive overview of the 1-year lifestyle profile and the cardiometabolic outcome framework, showing category distributions, primary endpoint frequencies, baseline cardiometabolic burden, condition-specific prevalence and incidence, and the distinction between the primary and secondary endpoints.

## 4. Discussion

This secondary analysis examined whether a pragmatic 1-year lifestyle profile was associated with the subsequent accumulation of common cardiometabolic risk factors after TBI. In the primary adjusted model, each additional favorable lifestyle behavior was associated with lower odds of developing at least two newly incident common cardiometabolic conditions between years 1 and 5. The estimate remained directionally stable after adjustment for the 1-year FIM cognitive score, in Firth bias-reduced regression, and in the ordinal sensitivity analysis. However, the primary model included only 38 events, the confidence interval approached the null, and the final cohort was a selected observed-data subset. The findings should therefore be interpreted as exploratory associations rather than as evidence of causality or as the validation of a prediction tool.

The descriptive results support the clinical relevance of cardiometabolic surveillance after TBI. Although the cohort was relatively young and most participants had two or more favorable lifestyle behaviors at 1 year, common cardiometabolic conditions accumulated over the subsequent 4-year interval. Hypertension and high cholesterol became more frequent by year 5, and more than one quarter of the primary at-risk cohort developed at least one new common cardiometabolic condition. These findings suggest that the period between the 1-year and 5-year follow-up assessments may represent an important window for prevention-oriented monitoring after inpatient rehabilitation.

The component-level analyses should be interpreted cautiously. Daily smoking and the absence of sports or exercise showed the clearest adverse descriptive and model-based signals, whereas alcohol category and BMI showed less precise patterns. These findings are hypothesis-generating because event counts within several component categories were sparse. They do not establish that smoking and exercise are the only relevant domains, nor do they imply that the four lifestyle components have equivalent biological effects. Rather, the cumulative lifestyle count provides a simple summary of observable behaviors, while component analyses help identify domains that may deserve particular attention in future studies and clinical follow-up.

### 4.1. Interpretation in the Context of Current Evidence

These results are consistent with the broader view of TBI as a chronic condition requiring long-term medical surveillance, not only neurological rehabilitation. Prior population-based studies have linked TBI with later medical comorbidity, including cardiometabolic outcomes, and the rehabilitation-focused literature increasingly emphasizes the need for chronic disease prevention in TBI survivorship care [53,54,55]. The present analysis adds a narrower observation: within a rehabilitation-enrolled TBI cohort, lifestyle behaviors measured at a routine 1-year follow-up were associated with the later accumulation of common cardiometabolic risk factors.

The findings should not be interpreted as evidence that the favorable lifestyle count is a TBI-specific risk score. Smoking, alcohol use, adiposity, and physical activity are general health domains, not TBI-specific mechanisms. Their relevance after TBI may reflect both general cardiometabolic pathways and the distinctive recovery context of TBI, including fatigue, motor limitations, cognitive inefficiency, emotional symptoms, reduced participation, transportation barriers, and changes in social roles. The stability of the estimate after adding the 1-year FIM cognitive score suggests that the association was not explained solely by broad cognitive functional status, but residual confounding remains possible.

The modeling strategy was intentionally parsimonious because the primary endpoint was uncommon. Sparse data bias and separation are important concerns in logistic regression when event counts are limited relative to the number of parameters [56,57,58,59,60]. For this reason, the main model used a restricted covariate set, the functional form of age was checked, and Firth bias-reduced regression was included as a sensitivity analysis. The predicted probability curve improved clinical interpretability by translating the adjusted odds ratio into absolute risk terms, but it should not be used for individual risk prediction. Similarly, the ordinal analysis supported the direction of the primary finding but remains a sensitivity analysis rather than a replacement for the prespecified binary endpoint.

### 4.2. Strengths, Limitations, and Clinical Implications

This study has several strengths. It used a national longitudinal TBI rehabilitation database, preserved temporal ordering by measuring lifestyle behaviors at 1 year and outcomes at 5 years, and focused the primary endpoint on common cardiometabolic risk factor accumulation rather than a heterogeneous mixture of risk factors and vascular events. The analysis also separated self-rated general health from observable lifestyle behaviors, documented cohort construction transparently, and used sensitivity analyses to address sparse event and injury severity coding issues.

The limitations are substantial. First, the final analytic cohort was a selected observed-data subset. Participants required valid 1-year lifestyle data, paired year-1/year-5 cardiometabolic ascertainment, baseline at-risk status, and complete covariates for adjusted modeling. These restrictions may introduce selection bias and limit generalizability to the broader TBIMS population, to TBI survivors followed only by proxy, and to individuals with TBI who did not receive inpatient rehabilitation. Second, cardiometabolic conditions were based on self-reported physician diagnoses rather than adjudicated clinical events, laboratory values, or medication records. Third, residual confounding is likely. Diet quality, sleep patterns, medication adherence, depressive symptoms, related neuropsychiatric sequelae, socioeconomic context, access to primary care, and health surveillance intensity were not modeled directly. Depression and other post-TBI neuropsychiatric symptoms are particularly relevant because they may influence lifestyle behaviors, follow-up engagement, and cardiometabolic risk. Fourth, although GCS- and PTA-adjusted sensitivity analyses were performed, robust severity-stratified analyses were not feasible because of sparse primary events and special or missing injury severity codes. Future studies with larger event counts should test whether injury severity modifies the association between lifestyle behaviors and cardiometabolic risk accumulation. Finally, this study was not designed to evaluate calibration, discrimination, decision thresholds, or prognostic performance of the lifestyle count.

The clinical implication is therefore pragmatic rather than predictive. The results support incorporating cardiometabolic prevention into routine post-TBI follow-up, particularly around the 1-year transition from early recovery to longer-term survivorship care. Follow-up visits should include the documentation of smoking status and cessation needs, the assessment of physical activity and exercise barriers, the monitoring of BMI or weight trajectory, blood pressure measurement, glucose or diabetes screening, and lipid status review. When indicated, referral pathways should include primary care, cardiology, rehabilitation, nutrition, behavioral health, adapted exercise programs, and smoking cessation services. The favorable lifestyle count may be useful as a simple communication aid, but it should not be used as a validated prediction tool or stand-alone risk stratification instrument.

Future studies should test these findings in larger and less selected cohorts, incorporate repeated lifestyle measurement, include richer TBI-specific severity and sequela measures, evaluate depressive and other neuropsychiatric symptoms, and use clinically adjudicated cardiometabolic outcomes when possible. Until such evidence is available, the most defensible conclusion is that observable lifestyle behaviors at 1 year after TBI appear relevant to later cardiometabolic risk factor accumulation and should be considered within prevention-oriented rehabilitation follow-up.

## 5. Conclusions

In this secondary analysis of the TBIMS National Database, a more favorable one-year lifestyle behavior profile was associated with lower odds of accumulating common cardiometabolic risk factors by five years after TBI. The association remained directionally stable across the main adjusted model, a model additionally accounting for one-year cognitive functional status, a Firth bias-reduced sensitivity analysis designed for sparse event settings, and an ordinal sensitivity model of incident burden. These results should be interpreted as exploratory associations in a selected observed-data cohort, not as evidence of causality, not as validation of a TBI-specific prognostic score, and not as support for using the four-behavior count as a stand-alone risk stratification instrument. The analysis supports a narrower and more defensible conclusion: within a rehabilitation-enrolled TBI cohort, post-injury smoking, exercise, alcohol pattern, and body weight status appear relevant to later cardiometabolic risk factor accumulation and deserve systematic attention during longitudinal follow-up.

This study also illustrates several methodological lessons. Clinically heterogeneous composite outcomes can weaken interpretation even when they appear to offer more events, structurally unavailable follow-up variables should not be handled as though they were ordinary missing values, and injury severity fields such as the GCS and PTA require explicit coding choices that preserve informative special states. The added ordinal analysis and probability visualization are useful for interpretation, but the central methodological stance remains conservative: transparent cohort construction, parsimonious modeling, the complete reporting of sparse event limitations, and the avoidance of overclaiming. Future work should test these findings in larger and less selected cohorts, incorporate repeated behavior measurement, and evaluate richer TBI-specific sequelae alongside adjudicated cardiometabolic outcomes. Until such evidence is available, the most clinically useful implication is pragmatic: one-year TBI follow-up should include attention to cardiometabolic prevention, especially smoking cessation and physical activity promotion, while broader medical surveillance remains integrated with long-term rehabilitation care.

## Figures and Tables

**Table 1:** The characteristics of the primary analytic cohort overall and by primary endpoint status. This table describes the adult primary at-risk cohort used for the main analysis. It reports baseline and one-year characteristics overall and separately for participants who did and did not experience the primary endpoint, allowing the reader to understand the clinical profile of the analytic sample and the distribution of potential confounders.

Characteristic	Overall (N = 581)	No Primary Endpoint (n = 542)	Primary Endpoint (n = 39)
Age at injury, mean (SD)	39.8 (17.7)	39.1 (17.4)	49.2 (18.9)
Female, n (%)	146 (25.1)	137 (25.3)	9 (23.1)
Race/ethnicity: White, n (%)	364 (62.7)	343 (63.3)	21 (53.8)
Race/ethnicity: Black, n (%)	102 (17.6)	95 (17.5)	7 (17.9)
Race/ethnicity: Hispanic, n (%)	90 (15.5)	81 (14.9)	9 (23.1)
Race/ethnicity: Other, n (%)	25 (4.3)	23 (4.2)	2 (5.1)
Education: ≤High school/trade, n (%)	297 (51.1)	277 (51.1)	20 (51.3)
Education: Some college/associate, n (%)	158 (27.2)	148 (27.3)	10 (25.6)
Education: Bachelor’s or higher, n (%)	123 (21.2)	115 (21.2)	8 (20.5)
Education: Unknown, n (%)	3 (0.5)	2 (0.4)	1 (2.6)
GCS category: Severe, n (%)	159 (27.4)	152 (28.0)	7 (17.9)
GCS category: Moderate, n (%)	60 (10.3)	57 (10.5)	3 (7.7)
GCS category: Mild, n (%)	163 (28.1)	149 (27.5)	14 (35.9)
GCS category: Intubated, n (%)	2 (0.3)	2 (0.4)	0 (0.0)
GCS category: Missing, n (%)	197 (33.9)	182 (33.6)	15 (38.5)
PTA duration: 0 days, n (%)	37 (6.4)	32 (5.9)	5 (12.8)
PTA duration: 1–7 days, n (%)	106 (18.2)	102 (18.8)	4 (10.3)
PTA duration: 8–28 days, n (%)	224 (38.6)	211 (38.9)	13 (33.3)
PTA duration: >28 days, n (%)	135 (23.2)	124 (22.9)	11 (28.2)
PTA duration: Still in PTA at discharge, n (%)	74 (12.7)	69 (12.7)	5 (12.8)
PTA duration: Unknown, n (%)	5 (0.9)	4 (0.7)	1 (2.6)
1-year FIM cognitive score, mean (SD)	30.6 (5.4)	30.7 (5.4)	29.6 (4.7)
Baseline common cardiometabolic burden: 0 conditions, n (%)	423 (72.8)	391 (72.1)	32 (82.1)
Baseline common cardiometabolic burden: 1 condition, n (%)	158 (27.2)	151 (27.9)	7 (17.9)

**Notes:** The primary at-risk cohort included adults aged 18 years or older with followed interviews at both 1 and 5 years, complete one-year four-behavior exposure data, complete common cardiometabolic outcome ascertainment at both waves, and fewer than two common conditions at year 1. Percentages are column percentages. **Abbreviations:** GCS, Glasgow Coma Scale; PTA, post-traumatic amnesia; FIM, Functional Independence Measure; SD, standard deviation.

**Table 2:** One-year exposure distributions and common cardiometabolic frequencies. This table summarizes the one-year lifestyle exposures in the primary at-risk cohort and the observed frequency of the primary endpoint within each category. It also reports baseline common cardiometabolic burden and the prevalence or incidence of the three common cardiometabolic conditions used to define the final outcome hierarchy.

Domain	Category	Distribution or Frequency	Primary Endpoint n/N (%)
**Panel A. One-year exposure distributions in the primary at-risk cohort**			
Current smoking at 1 year	Not at all	456 (78.5)	26/456 (5.7)
	Some days	34 (5.9)	2/34 (5.9)
	Every day	91 (15.7)	11/91 (12.1)
Alcohol category at 1 year	Light	128 (22.0)	6/128 (4.7)
	Abstaining	348 (59.9)	23/348 (6.6)
	Moderate/heavy	105 (18.1)	10/105 (9.5)
BMI category at 1 year	Under/normal	269 (46.3)	14/269 (5.2)
	Overweight	204 (35.1)	17/204 (8.3)
	Obesity	108 (18.6)	8/108 (7.4)
Sports/exercise frequency at 1 year	≥10/mo	220 (37.9)	10/220 (4.5)
	1–9/mo	171 (29.4)	8/171 (4.7)
	None	190 (32.7)	21/190 (11.1)
Favorable lifestyle count	0–1	24 (4.1)	1/24 (4.2)
	2	126 (21.7)	13/126 (10.3)
	3	288 (49.6)	21/288 (7.3)
	4	143 (24.6)	4/143 (2.8)
**Panel B. Common cardiometabolic burden and interval outcomes**			
Baseline common burden in at-risk cohort	0 conditions at year 1	423 (72.8)	32/423 (7.6)
	1 condition at year 1	158 (27.2)	7/158 (4.4)
Hypertension	Present at year 1	215/689 (31.2)	
	Present at year 5	308/689 (44.7)	
	Incident between year 1 and year 5	96/474 (20.3)	
Diabetes/high blood sugar	Present at year 1	56/689 (8.1)	
	Present at year 5	93/689 (13.5)	
	Incident between year 1 and year 5	37/633 (5.8)	
High cholesterol	Present at year 1	134/689 (19.4)	
	Present at year 5	218/689 (31.6)	
	Incident between year 1 and year 5	86/555 (15.5)	
Primary endpoint in at-risk cohort	≥2 new conditions	39/581 (6.7)	
Secondary endpoint in at-risk cohort	≥1 new condition	162/581 (27.9)	

**Notes:** Panel A reports one-year exposure distributions in the primary at-risk cohort and the corresponding primary endpoint frequency within each category. Panel B summarizes baseline common cardiometabolic burden in the primary at-risk cohort and the prevalence or incidence of the three common cardiometabolic conditions in the broader 689-participant cohort with complete year-1 and year-5 ascertainment. Descriptive endpoint counts in Table 2 are based on the full at-risk cohort (N = 581); adjusted model counts shown elsewhere use model-specific complete case samples and therefore may differ slightly. Abbreviations: BMI, body mass index.

**Table 3:** The main regression models for the one-year favorable lifestyle count. This table presents the main logistic regression models for the favorable lifestyle count, including the unadjusted estimate, the parsimonious adjusted models, the bias-reduced sparse data sensitivity analysis, and the broader six-condition sensitivity endpoint.

Outcome	Model	N	Events	OR per +1 Favorable Behavior (95% CI)	*p*
Primary endpoint: ≥2 newly incident common conditions	Model 1: unadjusted	577	38	0.72 (0.48–1.07)	0.100
	Model 2: + age, sex, race/ethnicity, education, baseline burden	577	38	0.63 (0.41–0.98)	0.040
	Model 3: Model 2 + 1-year FIM cognitive score	565	37	0.64 (0.41–0.99)	0.047
	Firth sensitivity: Model 2 covariates	577	38	0.64 (0.42–0.97)	0.037
Secondary endpoint: ≥1 newly incident common condition	Model 2: + age, sex, race/ethnicity, education, baseline burden	577	160	0.74 (0.58–0.95)	0.020
	Model 3: Model 2 + 1-year FIM cognitive score	565	155	0.73 (0.56–0.94)	0.016
Sensitivity endpoint: original 6-condition composite	Adjusted model with baseline 6-condition burden	680	53	0.76 (0.52–1.10)	0.143

Notes: Odds ratios are expressed per one additional favorable lifestyle behavior at year 1. For direct comparability, the unadjusted estimate was calculated in the same complete case sample as Model 2. Model 2 adjusted for age at injury, sex, race or ethnicity, education, and baseline common cardiometabolic burden; Model 3 additionally adjusted for the year-1 FIM cognitive score. The six-condition sensitivity model additionally adjusted for baseline six-condition burden. Model denominators differ because complete case requirements were model-specific. **Abbreviations:** CI, confidence interval; FIM, Functional Independence Measure.

**Table 4:** Component-level and self-rated general health analyses. This table reports the component-level analyses underlying the favorable lifestyle count and the separate analyses of self-rated general health. It clarifies whether the cumulative behavior count masks substantial heterogeneity across domains and addresses the conceptual decision to keep general health separate from the primary exposure.

Domain	Contrast or Subset	Adjusted OR (95% CI)	*p*
**Panel A. Component-level adjusted models for the primary endpoint**			
Smoking (ref: not at all)	N = 577, events = 38		
	Some days	1.21 (0.25–5.81)	0.816
	Every day	3.33 (1.38–8.02)	0.007
Alcohol (ref: light)	N = 577, events = 38		
	Abstaining	1.05 (0.40–2.78)	0.915
	Moderate/heavy	2.10 (0.71–6.17)	0.178
BMI (ref: under/normal)	N = 577, events = 38		
	Overweight	1.86 (0.84–4.12)	0.127
	Obesity	1.72 (0.66–4.48)	0.269
Exercise (ref: ≥10/mo)	N = 577, events = 38		
	1–9/mo	1.07 (0.40–2.84)	0.899
	None	2.45 (1.06–5.68)	0.037
**Panel B. Self-rated general health analyses**			
General health alone (ref: excellent/very good)	N = 486, events = 28		
	Good	2.91 (1.04–8.13)	0.042
	Fair/poor	4.07 (1.37–12.11)	0.012
Joint model (same subset)	N = 486, events = 28		
	Favorable lifestyle count per +1 point	0.82 (0.48–1.39)	0.463
	Good vs excellent/very good	2.78 (0.99–7.83)	0.053
	Fair/poor vs excellent/very good	3.71 (1.22–11.33)	0.021

Notes: Panel A reports component-level adjusted models for the primary endpoint using the same covariates as the primary adjusted model. Panel B reports analyses of self-rated general health on the restricted subset with valid general health data at year 1. Component-level estimates are exploratory and hypothesis-generating because the number of primary events was limited. **Abbreviations:** BMI, body mass index; CI, confidence interval.

## Data Availability

The data that support the findings of this study are available from the Traumatic Brain Injury Model Systems National Data and Statistical Center (TBIMS NDSC). De-identified data can be accessed by qualified investigators upon the completion of a Data Use Agreement through the TBIMS NDSC. The authors are not permitted to redistribute the dataset directly.

## Supplementary Materials

The following supporting information can be downloaded at: https://www.mdpi.com/article/10.3390/medsci14020265/s1, Supplementary Materials S1–S8. Supplementary Material S1: Cohort assembly and stepwise attrition. Supplementary Material S2: Included-versus-excluded comparisons after age and follow-up eligibility. Supplementary Material S3: Variable codebook; analytic recoding rules for primary exposures, outcomes, and covariates; and reproducibility pseudocode. Supplementary Material S4: Missing data structure, structural unavailability, and completeness by variable and wave. Supplementary Material S5: Design analysis and sparse event contextualization, including age form assessment and model capacity justification. Supplementary Material S6: Robustness analyses for primary exposure and endpoint, including alternative handling of Glasgow Coma Scale and post-traumatic amnesia variables. Supplementary Material S7: Outcome-specific models, alternative endpoint analyses, ordinal logistic regression, and model-based predicted probabilities. Supplementary Material S8: STROBE checklist.
