# Supplementary material for: The First Year Matters: Lifestyle Behaviors and Five-Year Cardiometabolic Risk Factor Accumulation After Traumatic Brain Injury
Source: Med Sci (Basel). 2026 May 20;14(2):265. doi: 10.3390/medsci14020265 (PMC13214714; doi:10.3390/medsci14020265)
Supplement: Supplementary file 1 [file medsci-14-00265-s001.zip › Supplementary Material 1.docx]

**Supplementary Material 1. Cohort Assembly and Stepwise Attrition**. This supplementary material documents each step used to derive the final adult analytic cohort from the original linked TBIMS files. It is essential for understanding how observed-data restrictions, rather than arbitrary pruning, shaped the final sample.

| **Cohort step** | **N retained** | **Reason for attrition** |
| --- | --- | --- |
| TBIMS Form 1 records in the public-use dataset | 20167 | Initial baseline records |
| Linked to followed 1-year and 5-year Form 2 interviews | 10057 | Excluded if not followed at one or both target waves or not linkable by Mod1Id |
| Age >=18 years at injury | 9593 | Excluded ages 16-17 years or missing age at injury |
| Complete one-year four-behavior exposure | 3182 | Excluded for structural unavailability or invalid smoking, alcohol, BMI, or exercise data |
| Complete one-year and five-year common cardiometabolic outcomes | 689 | Excluded for incomplete hypertension, diabetes/high blood sugar, or high-cholesterol status at either wave |
| Primary at-risk cohort | 581 | Excluded prevalent baseline burden >=2 common conditions at year 1 |
| Primary adjusted-model complete-case sample | 577 | Excluded 4 participants with missing sex or education |
| Primary adjusted model plus one-year FIM cognitive score | 565 | Excluded 12 additional participants with missing year-1 FIM cognitive score |

*Notes: The cohort assembly begins with all Form 1 records and then applies linkage, age restriction, observed exposure and outcome requirements, and final covariate completeness. Abbreviations: TBIMS, Traumatic Brain Injury Model Systems; FIM, Functional Independence Measure.*
