# Supplementary material for: The First Year Matters: Lifestyle Behaviors and Five-Year Cardiometabolic Risk Factor Accumulation After Traumatic Brain Injury
Source: Med Sci (Basel). 2026 May 20;14(2):265. doi: 10.3390/medsci14020265 (PMC13214714; doi:10.3390/medsci14020265)
Supplement: Supplementary file 1 [file medsci-14-00265-s001.zip › Supplementary Material 2.docx]

**Supplementary Material 2. Comparison of Included and Excluded Eligible Participants**. This supplementary material compares the final primary at-risk cohort with other age-eligible participants who had the required follow-up waves but were excluded later during observed-data cohort construction. The table helps readers judge the direction and magnitude of potential selection patterns.

| **Characteristic** | **Included (n=581)** | **Excluded (n=9012)** | **Absolute SMD** |
| --- | --- | --- | --- |
| Age at injury, mean (SD) | 39.8 (17.7) | 40.7 (17.8) | 0.05 |
| Female, n (%) | 146 (25.1) | 2443 (27.1) | 0.05 |
| Race/ethnicity: White, n (%) | 364 (62.7) | 6235 (69.2) | 0.14 |
| Race/ethnicity: Black, n (%) | 102 (17.6) | 1567 (17.4) | 0.00 |
| Race/ethnicity: Hispanic, n (%) | 90 (15.5) | 820 (9.1) | 0.19 |
| Race/ethnicity: Other, n (%) | 25 (4.3) | 390 (4.3) | 0.00 |
| Education: <=High school/trade, n (%) | 297 (51.1) | 4971 (55.2) | 0.08 |
| Education: Some college/associate, n (%) | 158 (27.2) | 2356 (26.1) | 0.02 |
| Education: Bachelor's or higher, n (%) | 123 (21.2) | 1601 (17.8) | 0.09 |
| Education: Unknown, n (%) | 3 (0.5) | 84 (0.9) | 0.05 |
| GCS category: Severe, n (%) | 159 (27.4) | 3151 (35.0) | 0.16 |
| GCS category: Moderate, n (%) | 60 (10.3) | 1025 (11.4) | 0.03 |
| GCS category: Mild, n (%) | 163 (28.1) | 2632 (29.2) | 0.03 |
| GCS category: Intubated, n (%) | 2 (0.3) | 18 (0.2) | 0.03 |
| GCS category: Missing, n (%) | 197 (33.9) | 2186 (24.3) | 0.21 |
| 1-year FIM cognitive score, mean (SD) | 30.6 (5.4) | 30.6 (5.2) | 0.01 |

*Notes: Included participants are those in the primary at-risk cohort. Excluded participants are age-eligible and follow-up-eligible adults who were later excluded during observed-data cohort construction. Standardized mean differences are absolute values. Abbreviations: GCS, Glasgow Coma Scale; FIM, Functional Independence Measure; PTA, post-traumatic amnesia; SD, standard deviation; SMD, standardized mean difference.*
