# Supplementary material for: The First Year Matters: Lifestyle Behaviors and Five-Year Cardiometabolic Risk Factor Accumulation After Traumatic Brain Injury
Source: Med Sci (Basel). 2026 May 20;14(2):265. doi: 10.3390/medsci14020265 (PMC13214714; doi:10.3390/medsci14020265)
Supplement: Supplementary file 1 [file medsci-14-00265-s001.zip › Supplementary Material 3.docx]

**Supplementary Material 3. Analytic Codebook and Recoding Rules**. This supplementary material provides the analytic codebook for all exposures, outcomes, and covariates used in the final manuscript. It documents the source fields, recoding logic, and treatment of special TBIMS codes so that the final analysis can be reproduced transparently.

| **Analytic variable** | **Source form** | **Original field(s)** | **Analytic coding rule** | **Role in analysis** |
| --- | --- | --- | --- | --- |
| age_years | Form 1 | AGENoPHI | Continuous age at injury; 777 treated as 89 years or older; 999 and implausible negatives treated as missing | Covariate |
| sex_cat | Form 1 | SexF | Female, male; code 99 treated as missing in primary adjusted models | Covariate |
| race_cat | Form 1 | Race | Collapsed to White, Black, Hispanic, and Other; unknown/not applicable grouped with Other only descriptively and treated as Other in tables | Covariate / descriptive |
| education_cat | Form 1 | EDUCATION | Collapsed to <=High school/trade, Some college/associate, and Bachelor's or higher; unknown treated as missing in primary adjusted models | Covariate |
| gcs_cat | Form 1 | GCSCat | Severe, moderate, mild, intubated, missing; note that the public CSV stores intubated as 8 and missing as 99 although auxiliary code files list 77 and 999 | Robustness covariate |
| pta_cat | Form 1 | PTADays | 0 days, 1-7 days, 8-28 days, >28 days, still in PTA at rehabilitation discharge, unknown | Robustness covariate |
| FIMCOGF_valid | Form 2 year 1 | FIMCOGF | Continuous one-year FIM Cognitive score; 999 treated as missing | TBI-context robustness covariate |
| SmkCigF | Form 2 year 1 | SmkCigF | Valid codes 1-3; favorable component defined as not at all | Exposure component |
| DRINKCatF | Form 2 year 1 | DRINKCatF | Valid codes 0-3; favorable component defined as abstaining/light/moderate | Exposure component |
| BMICatF | Form 2 year 1 | BMICatF | Valid codes 1-8; favorable component defined as underweight/normal/overweight | Exposure component |
| PRTPlaySportF | Form 2 year 1 | PRTPlaySportF | Valid codes 0-5; favorable component defined as >=10 times per month | Exposure component |
| lifestyle_count | Derived | SmkCigF + DRINKCatF + BMICatF + PRTPlaySportF | Sum of four favorable lifestyle indicators, range 0-4 | Primary exposure |
| GenHlthF | Form 2 year 1 | GenHlthF | Valid codes 1-5 retained only for separate and joint analyses; 66, 82, and 99 treated as unavailable | Secondary explanatory exposure |
| HypertensionF | Form 2 years 1 and 5 | HypertensionF | Valid yes/no at both waves required for endpoint derivation | Outcome component |
| DiabetesHighBloodSugarF | Form 2 years 1 and 5 | DiabetesHighBloodSugarF | Valid yes/no at both waves required for endpoint derivation | Outcome component |
| HighBloodCholesterolF | Form 2 years 1 and 5 | HighBloodCholesterolF | Valid yes/no at both waves required for endpoint derivation | Outcome component |
| primary_event | Derived | HypertensionF + DiabetesHighBloodSugarF + HighBloodCholesterolF | At least two new common cardiometabolic conditions between years 1 and 5 among participants with <=1 condition at year 1 | Primary endpoint |
| secondary_event | Derived | Same three-condition set | At least one new common cardiometabolic condition between years 1 and 5 among participants with <=1 condition at year 1 | Secondary endpoint |

*Notes: The codebook summarizes the variables used in the final analysis, their source forms, recoding rules, and treatment of special TBIMS codes. Abbreviations: BMI, body mass index; FIM, Functional Independence Measure; GCS, Glasgow Coma Scale; PTA, post-traumatic amnesia; TBIMS, Traumatic Brain Injury Model Systems.*
