# Supplementary material for: The First Year Matters: Lifestyle Behaviors and Five-Year Cardiometabolic Risk Factor Accumulation After Traumatic Brain Injury
Source: Med Sci (Basel). 2026 May 20;14(2):265. doi: 10.3390/medsci14020265 (PMC13214714; doi:10.3390/medsci14020265)
Supplement: Supplementary file 1 [file medsci-14-00265-s001.zip › Supplementary Material 4.docx]

**Supplementary Material 4. Wave-Specific Completeness and Structural Unavailability**. This supplementary material details how much usable information was available for the one-year exposure variables and for paired year-1/year-5 outcome ascertainment. It highlights the distinction between complete data and non-complete data created by structural unavailability, invalid codes, or interview constraints.

*Panel A. One-year exposure completeness in the adult linked cohort*

| **Variable at 1 year** | **Complete n** | **Denominator** | **Complete %** |
| --- | --- | --- | --- |
| Current cigarette smoking at 1 year | 3329 | 9593 | 34.7 |
| Alcohol category at 1 year | 8914 | 9593 | 92.9 |
| BMI category at 1 year | 3251 | 9593 | 33.9 |
| Sports/exercise frequency at 1 year | 5977 | 9593 | 62.3 |
| Self-rated general health at 1 year | 2725 | 9593 | 28.4 |

*Panel B. Paired year-1/year-5 outcome completeness among participants with complete four-behavior exposure*

| **Outcome pair complete at 1 and 5 years** | **Complete n** | **Denominator** | **Complete %** |
| --- | --- | --- | --- |
| Hypertension | 709 | 3182 | 22.3 |
| Diabetes/high blood sugar | 706 | 3182 | 22.2 |
| High cholesterol | 692 | 3182 | 21.7 |
| Heart attack | 701 | 3182 | 22.0 |
| Congestive heart failure | 703 | 3182 | 22.1 |
| Stroke | 702 | 3182 | 22.1 |

*Notes: Completeness is shown first for the one-year exposure variables in the adult linked cohort and then for paired year-1/year-5 outcome ascertainment among participants with complete four-behavior exposure data. Structural unavailability and invalid special codes contribute importantly to non-completeness.*
