# Supplementary material for: The First Year Matters: Lifestyle Behaviors and Five-Year Cardiometabolic Risk Factor Accumulation After Traumatic Brain Injury
Source: Med Sci (Basel). 2026 May 20;14(2):265. doi: 10.3390/medsci14020265 (PMC13214714; doi:10.3390/medsci14020265)
Supplement: Supplementary file 1 [file medsci-14-00265-s001.zip › Supplementary Material 5.docx]

**Supplementary Material 5. Design Analysis, Sparse-Event Contextualization, and Age-Form Assessment**. This supplementary material contextualizes the sparse-event environment of the primary adjusted model. It reports sample size, event counts, model capacity, the age non-linearity assessment, and a descriptive detectable-effect calculation to support methodologic transparency.

| **Design-analysis item** | **Result** |
| --- | --- |
| Primary at-risk cohort size | 581 participants |
| Primary endpoint frequency | 39/581 (6.7%) |
| Primary adjusted-model sample | 577 participants; 38 events |
| Model parameters in the primary adjusted model | 9 parameters excluding the intercept |
| Observed events per parameter | 38/9 = 4.2 |
| Age non-linearity likelihood-ratio test | p = 0.991 for spline versus linear age |
| Approximate 80% detectable odds ratio per +1 lifestyle point | approximately 0.56 (or 1.79 in the opposite direction) under the observed score distribution and event fraction |

*Notes: This design-analysis summary contextualizes the sparse-event setting of the primary adjusted model and reports the formal comparison of linear and spline age terms. The detectable-effect calculation is an approximate descriptive aid rather than a post hoc hypothesis test.*
