# Supplementary material for: The First Year Matters: Lifestyle Behaviors and Five-Year Cardiometabolic Risk Factor Accumulation After Traumatic Brain Injury
Source: Med Sci (Basel). 2026 May 20;14(2):265. doi: 10.3390/medsci14020265 (PMC13214714; doi:10.3390/medsci14020265)
Supplement: Supplementary file 1 [file medsci-14-00265-s001.zip › Supplementary Material 6.docx]

**Supplementary Material 6. Robustness Analyses for the Primary Exposure and Endpoint**. This supplementary material presents the main sensitivity analyses used to evaluate the stability of the primary estimate. It includes the FIM-adjusted model, Firth bias-reduced regression, the broader age sensitivity cohort, and alternative handling of GCS and PTA.

| **Analysis** | **N** | **Events** | **OR per +1 lifestyle point (95% CI)** | **p** |
| --- | --- | --- | --- | --- |
| Primary endpoint, main adjusted model | 577 | 38 | 0.63 (0.41-0.98) | 0.040 |
| Primary endpoint, FIM cognitive added | 565 | 37 | 0.64 (0.41-0.99) | 0.047 |
| Primary endpoint, Firth bias-reduced logistic | 577 | 38 | 0.64 (0.42-0.97) | 0.037 |
| Primary endpoint, age >=16 years sensitivity | 590 | 38 | 0.63 (0.41-0.97) | 0.037 |
| Primary endpoint, explicit GCS categories | 577 | 38 | 0.63 (0.41-0.98) | 0.041 |
| Primary endpoint, explicit PTA categories | 577 | 38 | 0.65 (0.42-1.00) | 0.052 |

*Notes: All robustness analyses evaluate the favorable lifestyle count against the primary endpoint unless otherwise stated. The explicit GCS and PTA models retain clinically informative severity categories rather than collapsing them into generic missingness. Abbreviations: CI, confidence interval; FIM, Functional Independence Measure; GCS, Glasgow Coma Scale; PTA, post-traumatic amnesia.*
