# Supplementary material for: The First Year Matters: Lifestyle Behaviors and Five-Year Cardiometabolic Risk Factor Accumulation After Traumatic Brain Injury
Source: Med Sci (Basel). 2026 May 20;14(2):265. doi: 10.3390/medsci14020265 (PMC13214714; doi:10.3390/medsci14020265)
Supplement: Supplementary file 1 [file medsci-14-00265-s001.zip › Supplementary Material 7.docx]

**Supplementary Material 7. Alternative Endpoint Models and Outcome-Specific Analyses**. This supplementary material reports alternative endpoint models and condition-specific analyses. It complements the main tables by showing how the favorable lifestyle count behaved for the secondary endpoint, the original six-condition composite, and the three individual common cardiometabolic outcomes. Because the condition-specific models were fit on endpoint-specific complete-case sets, their analytic denominators can differ slightly from the descriptive at-risk counts shown in the main tables.

*Panel A. Alternative endpoint models for the favorable lifestyle count*

| **Endpoint** | **N** | **Events** | **Adjusted OR per +1 lifestyle point (95% CI)** | **p** |
| --- | --- | --- | --- | --- |
| Secondary endpoint: >=1 incident common condition | 577 | 160 | 0.74 (0.58-0.95) | 0.020 |
| Secondary endpoint + FIM cognitive | 565 | 155 | 0.73 (0.56-0.94) | 0.016 |
| Original six-condition composite: >=2 incident conditions | 680 | 53 | 0.76 (0.52-1.10) | 0.143 |

*Panel B. Outcome-specific models for the common cardiometabolic conditions*

| **Endpoint** | **N at risk** | **Events** | **Adjusted OR per +1 lifestyle point (95% CI)** | **p** |
| --- | --- | --- | --- | --- |
| Incident hypertension | 484 | 99 | 0.81 (0.59-1.09) | 0.167 |
| Incident diabetes/high blood sugar | 643 | 38 | 0.53 (0.34-0.83) | 0.005 |
| Incident high cholesterol | 552 | 84 | 0.80 (0.58-1.10) | 0.175 |

*Notes: Panel A reports alternative endpoint models for the favorable lifestyle count. For Panel A, differences between descriptive and model-based event counts arise from model-specific complete-case restrictions rather than from redefinition of the endpoint. Panel B reports outcome-specific models for each common cardiometabolic condition among participants free of the corresponding condition at year 1; these exploratory models were adjusted for age, sex, race or ethnicity, and education. For Panel B, the reported N values reflect condition-specific complete-case model samples with valid endpoint ascertainment and covariates, so they may differ modestly from the descriptive at-risk denominators reported in Table 2. Abbreviations: CI, confidence interval; FIM, Functional Independence Measure.*
