# Supplementary material for: The First Year Matters: Lifestyle Behaviors and Five-Year Cardiometabolic Risk Factor Accumulation After Traumatic Brain Injury
Source: Med Sci (Basel). 2026 May 20;14(2):265. doi: 10.3390/medsci14020265 (PMC13214714; doi:10.3390/medsci14020265)
Supplement: Supplementary file 1 [file medsci-14-00265-s001.zip › Supplementary Material 8.docx]

Supplementary Material 8. STROBE Checklist Aligned to the Medical Sciences-Formatted Manuscript. This supplementary material provides the STROBE checklist aligned to the final Medical Sciences/MDPI manuscript and its rendered page numbers. It allows readers to verify where each reporting element is addressed in the final paper.

| **Item** | **Section topic** | **Recommendation** | **Manuscript section** | **Page(s)** |
| --- | --- | --- | --- | --- |
| 1 | Title and abstract | Indicate the study design with a commonly used term in the title or abstract. | Title; Abstract | 1-2 |
| 2 | Background/rationale | Explain the scientific background and rationale for the investigation. | Introduction | 2-4 |
| 3 | Objectives | State specific objectives and any prespecified hypotheses. | Introduction | 4 |
| 4 | Study design | Present key elements of study design early in the paper. | Materials and Methods | 4 |
| 5 | Setting | Describe the setting, locations, and relevant dates, including follow-up. | Materials and Methods | 4-5 |
| 6a | Participants | Give the eligibility criteria and the sources and methods of selection of participants. | Materials and Methods | 5-6 |
| 6b | Participants | Describe follow-up methods. | Materials and Methods | 4-5 |
| 7 | Variables | Clearly define all outcomes, exposures, predictors, confounders, and effect modifiers. | Materials and Methods | 6-9 |
| 8 | Data sources/measurement | For each variable of interest, give sources of data and details of methods of assessment. | Materials and Methods | 4, 6-10 |
| 9 | Bias | Describe efforts to address potential sources of bias. | Materials and Methods; Discussion | 8-10, 26-27 |
| 10 | Study size | Explain how the study size was arrived at. | Materials and Methods; Supplementary Material 5 | 5-6, 9-11 |
| 11 | Quantitative variables | Explain how quantitative variables were handled in the analyses. | Materials and Methods | 6-10 |
| 12a | Statistical methods | Describe all statistical methods, including those used to control for confounding. | Materials and Methods | 9-10 |
| 12b | Statistical methods | Describe methods used to examine subgroups and interactions. | Materials and Methods; Results | 6-7, 10, 16-22 |
| 12c | Statistical methods | Explain how missing data were addressed. | Materials and Methods | 8-9 |
| 12d | Statistical methods | If applicable, explain how loss to follow-up was addressed. | Materials and Methods; Supplementary Material 1 | 5, 11-12, 28 |
| 12e | Statistical methods | Describe any sensitivity analyses. | Materials and Methods; Results | 10, 16-22 |
| 13a | Participants | Report numbers of individuals at each stage of the study. | Results; Supplementary Material 1 | 11-12, 28 |
| 13b | Participants | Give reasons for non-participation at each stage. | Supplementary Material 1 | 12, 28 |
| 13c | Participants | Consider use of a flow diagram. | Supplementary Material 1 | 12 |
| 14a | Descriptive data | Give characteristics of study participants and information on exposures and confounders. | Results; Table 1; Table 2 | 12-16 |
| 14b | Descriptive data | Indicate the number of participants with missing data for each variable of interest. | Supplementary Material 4 | 8-10, 20 |
| 15 | Outcome data | Report numbers of outcome events or summary measures. | Results; Table 2 | 14-16, 23 |
| 16a | Main results | Give unadjusted and adjusted estimates with precision. | Results; Table 3 | 16-20 |
| 16b | Main results | Report category boundaries when continuous variables were categorized. | Materials and Methods; Table 2 | 6-7, 15-16 |
| 16c | Main results | If relevant, consider translating relative estimates into absolute risk. | Results; Table 2 | 14-16, 23 |
| 17 | Other analyses | Report other analyses done, such as subgroup, sensitivity, or outcome-specific analyses. | Results; Table 4; Supplementary Materials 6-7 | 16-22, 28 |
| 18 | Key results | Summarize key results with reference to study objectives. | Discussion | 23-25 |
| 19 | Limitations | Discuss limitations, taking into account sources of potential bias or imprecision. | Discussion | 26-27 |
| 20 | Interpretation | Give a cautious overall interpretation considering objectives, limitations, and evidence. | Discussion | 23-27 |
| 21 | Generalisability | Discuss the generalisability of the study results. | Discussion | 26-27 |
| 22 | Funding | Give the source of funding and the role of funders. | Funding | 28 |

Notes: The checklist is aligned to the final Medical Sciences/MDPI-formatted manuscript file and corresponding rendered page numbers. Page references correspond to the submission-style manuscript with figures and tables embedded in the main text near their first citation.
